# The identification of high-performing antibodies for TDP-43 for use in Western Blot, immunoprecipitation and immunofluorescence

**DOI:** 10.12688/f1000research.131852.2

**Published:** 2023-06-20

**Authors:** Donovan Worrall, Riham Ayoubi, Maryam Fotouhi, Kathleen Southern, Peter S. McPherson, Carl Laflamme, NeuroSGC/YCharOS/EDDU collaborative group, ABIF Consortium

**Affiliations:** 1 Department of Neurology and Neurosurgery, Structural Genomics Consortium, The Montreal Neurological Institute, McGill University, Montreal, Quebec, H3A 2B4, Canada

**Keywords:** Uniprot ID Q13148, TARDBP, TDP-43, antibody characterization, antibody validation, Western Blot, immunoprecipitation, immunofluorescence

## Abstract

TAR DNA-binding protein 43 (TDP-43) is a DNA/RNA binding protein playing a critical role in the regulation of transcription, splicing and RNA stability. Mutations in *TARDBP *leading to aggregation, are suspected to be a characteristic feature of various neurogenerative diseases. The lack of well-characterized anti- TDP-43 antibodies acts as a barrier to establish reproducible TDP-43 research. In this study, we characterized eighteen TDP-43 commercial antibodies for Western blot, immunoprecipitation, and immunofluorescence using a standardized experimental protocol based on comparing read-outs in knockout cell lines and isogenic parental controls. We identified many well-performing antibodies and encourage readers to use this report as a guide to select the most appropriate antibody for their specific needs.

## Introduction

TDP-43, encoded by the *TARDBP* gene, is a DNA/RNA-binding protein implicated in RNA metabolism and processing. ^1^ Belonging to the heterogeneous nuclear ribonucleoprotein (hnRNP) family of proteins that bind to RNA via highly conserved RNA recognition motifs, TDP-43 binds to UG-repeats with high specificity. ^1^^,^^2^

Mutations in *TARDBP* that result in TDP-43 aggregation and neuropathology have been observed in distinct neurodegenerative diseases, known as TDP-43 proteinopathies. ^3^^,^^4^ Various studies have identified a subset of amyotrophic lateral sclerosis (ALS) patients that possess *TARDBP* mutations, suggesting that TDP-43 gain of toxic function or loss of function is a causative factor in sporadic and/or familial ALS. ^4^^–^^6^ Mechanistic studies would be greatly facilitated by the availability of high-quality antibodies.

Here, we compared the performance of a range of commercially available antibodies for TDP-43 and characterized high-quality antibodies for Western blot, immunoprecipitation and immunofluorescence, enabling biochemical and cellular assessment of TDP-43 properties and function.

## Results and discussion

Our standard protocol involved comparing readouts from wild-type (WT) and *TARDBP* knockout (KO) cells. ^7^^,^^8^ The first step was to identify a cell line(s) that expresses sufficient levels of TDP-43 to generate a measurable signal. To this end, we examined the DepMap transcriptomics database to identify all cell lines that express the target at levels greater than 2.5 log _2_ (transcripts per million “TPM” +1), which we have found to be a suitable cut-off (Cancer Dependency Map Portal, RRID: SCR_017655). Commercially available HAP1 cells expressed the *TARDBP* transcript at RNA levels above the average range of cancer cells analyzed. Parental and *TARDBP* knockout HAP1 cells were obtained from Horizon Discovery ( Table 1).

**Table 1.** Summary of the cell lines used.

Institution	Catalog number	RRID (Cellosaurus)	Cell line	Genotype
Horizon Discovery	C631	CVCL_Y019	HAP1	WT
Horizon Discovery	HZGHC003730c001	CVCL_TR64	HAP1	*TADRBP* KO

**Figure 1.**
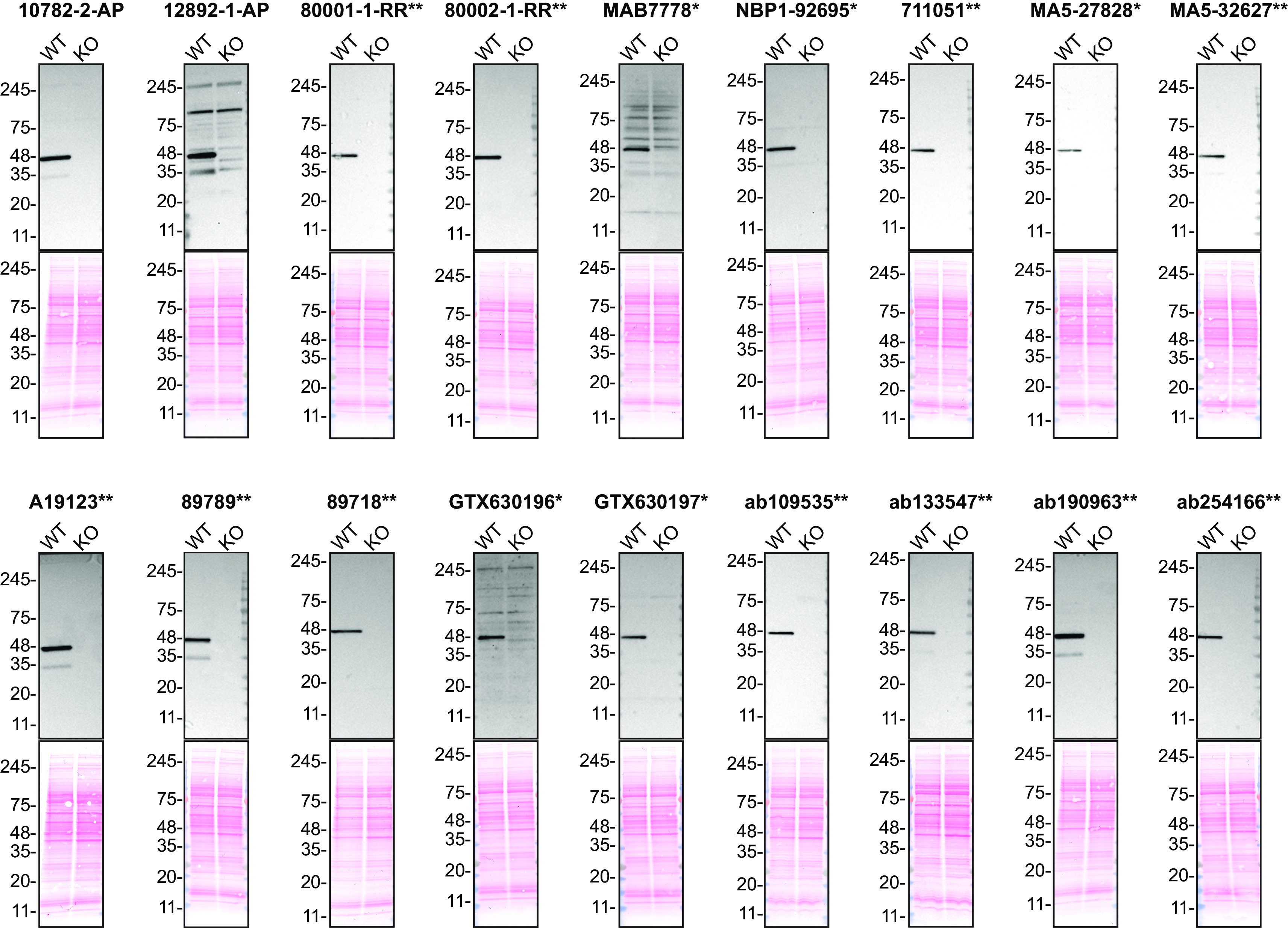
TDP-43 antibody screening by Western blot. Lysates of HAP1 (WT and *TARDBP* KO) were prepared and 50 μg of protein were processed for Western blot with the indicated TDP-43 antibodies. The Ponceau stained transfers of each blot are shown. Antibody dilutions were chosen according to the recommendations of the antibody supplier. Exceptions were given for antibody 80001-1-RR**, which was titrated to 1/1000 as the signal was too weak when following the supplier’s recommendations. When the concentration was not indicated by the supplier, which was the case for antibody 80002-1-RR**, we tested the antibody at 1/1000. Antibody dilution used: 10782-2-AP at 1/5000, 12892-1-AP at 1/1000, 800001-1-RR** at 1/1000, 80002-1-RR** at 1/1000, MAB7778* at 1/500, NBP1-92695* at 1/1000, 711051** at 1/1000, MA5-27828* at 1/1000, MA5-32627** at 1/1000, A19123** at 1/1000, 89789** at 1/1000, 89718** at 1/1000, GTX630196* at 1/500, GTX630197* at 1/500, ab109535** at 1/2000, ab133547** at 1/1000, ab190963** at 1/1000, ab254166** at 1/1000. Predicted band size: 45 kDa. *Monoclonal antibody, **Recombinant antibody.

For Western Blot experiments, we resolved proteins from WT and *TARDBP* KO cell extracts and probed them side-by-side with all antibodies in parallel ( Figure 1).

**Figure 2.**
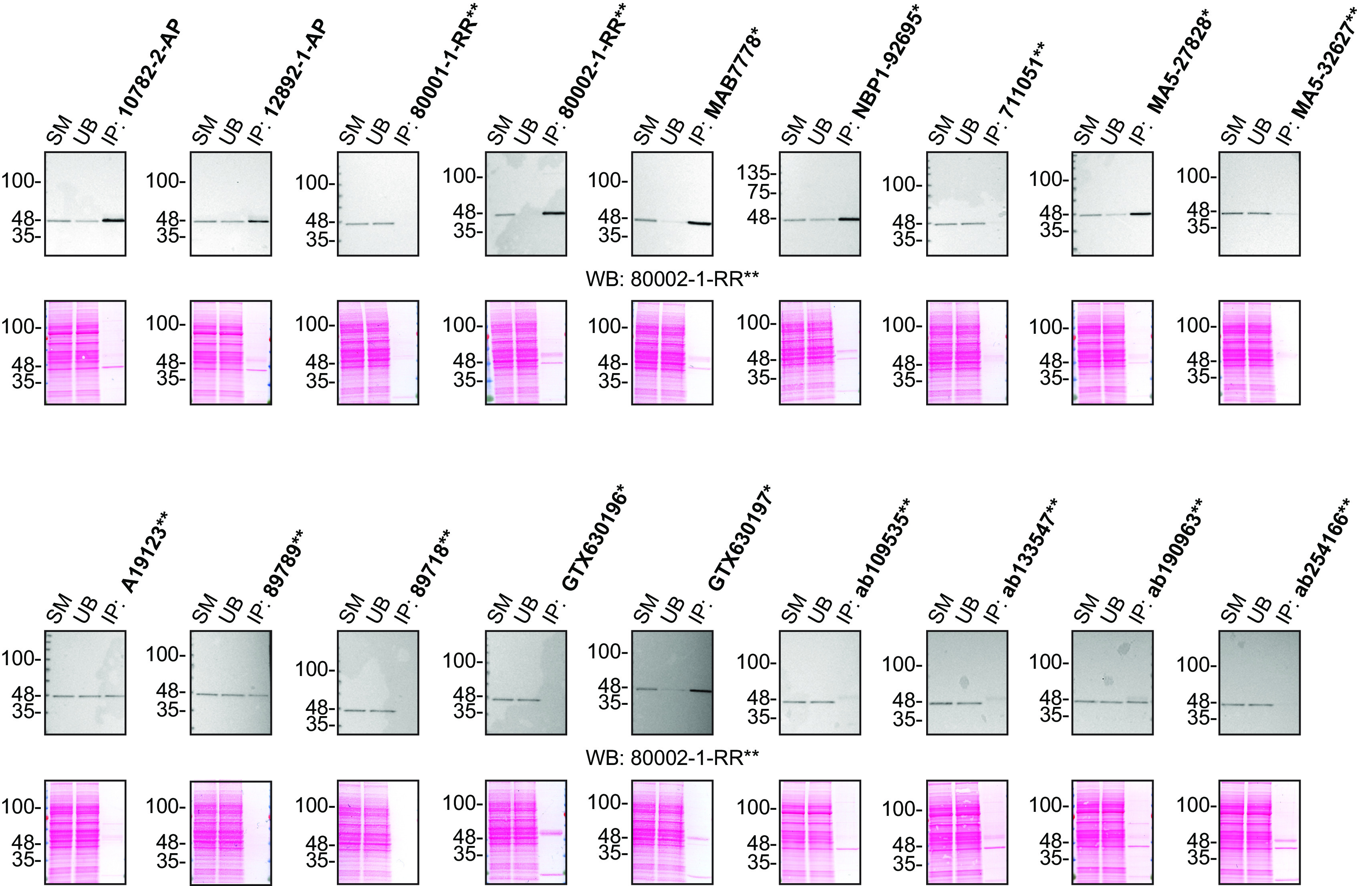
TDP-43 antibody screening by immunoprecipitation. HAP1 lysates were prepared, and IP was performed using 2.0 μg of the indicated TDP-43 antibodies pre-coupled to Dynabeads protein G or protein A. Samples were washed and processed for Western blot with the indicated TDP-43 antibody. For Western blot, 80002-1-RR** was used at 1/1000. The Ponceau stained transfers of each blot are shown for similar reasons as in Figure 1. SM=4% starting material; UB=4% unbound fraction; IP=immunoprecipitate. *Monoclonal antibody, **Recombinant antibody.

For immunoprecipitation experiments, we used the antibodies to immunopurify TDP-43 from HAP1 cell extracts. The performance of each antibody was evaluated by detecting the TDP-43 protein in extracts, in the immunodepleted extracts and in the immunoprecipitates ( Figure 2).

**Figure 3.**
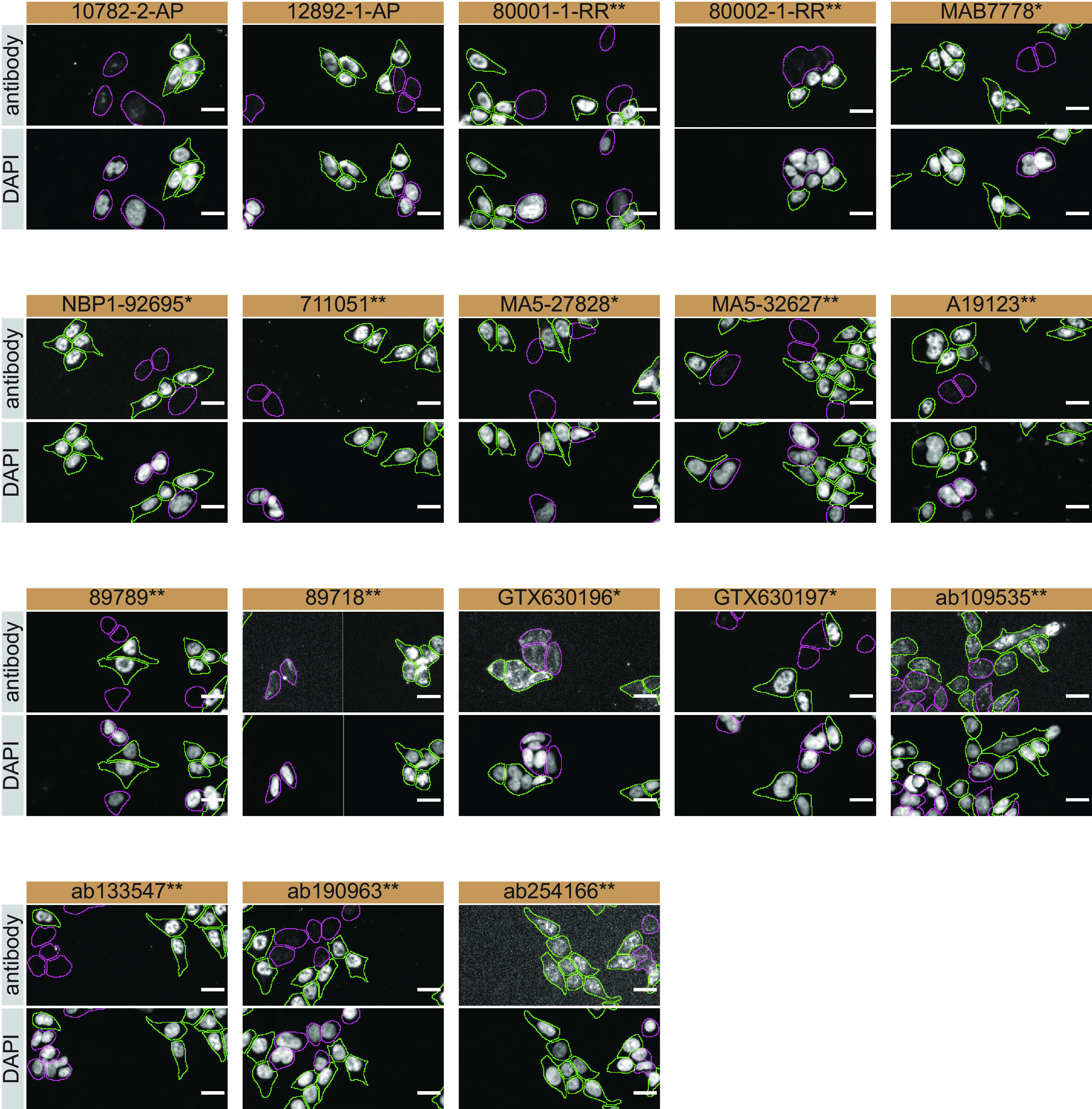
TDP-43 antibody screening by immunofluorescence. HAP1 WT and *TARDBP* KO cells were labelled with a green or a far-red fluorescent dye, respectively. WT and KO cells were mixed and plated to a 1:1 ratio in a 96-well plate with a glass bottom. Cells were stained with the indicated TDP-43 antibodies and with the corresponding Alexa-fluor 555 coupled secondary antibody including DAPI. Acquisition of the blue (nucleus-DAPI), green (identification of WT cells), red (antibody staining) and far-red (identification of KO cells) channels was performed. Representative images of the merged blue and red (grayscale) channels are shown. WT and KO cells are outlined on both channels with green and magenta dashed lines, respectively. Antibody dilutions were chosen according to the recommendations of the antibody supplier. Exceptions were given for antibodies 12892-1-AP, MA5-32627**, A19123**, 89789**, 89718** and ab133547** which were titrated to 1/400, 1/1000, 1/900, 1/30, 1/10 and 1/700, respectively, as the signals were too weak when following the supplier’s recommendations When the concentration was not indicated by the supplier, which was the case for antibodies 80001-1-RR**, GTX630196* and ab254166**, we tested antibodies at 1/700, 1/1000 and 1/500, respectively. At these concentrations, the signal from each antibody was in the range of detection of the microscope used. Antibody dilution used: 10782-2-AP at 1/400, 12892-1-AP at 1/250, 800001-1-RR** at 1/700, 80002-1-RR** at 1/250, MAB7778* at 1/500, NBP1-92695* at 1/1000, 711051** at 1/500, MA5-27828* at 1/1000, MA5-32627** at 1/1000, A19123** at 1/900, 89789** at 1/30, 89718** at 1/10, GTX630196* at 1/1000, GTX630197* at 1/1000, ab109535** at 1/30, ab133547** at 1/700, ab190963** at 1/800, ab254166** at 1/500. Bars = 10 μm. *Monoclonal antibody, **Recombinant antibody.

For immunofluorescence, as described previously, antibodies were screened using a mosaic strategy. ^9^ In brief, we plated WT and KO cells together in the same well and imaged both cell types in the same field of view to reduce staining, imaging and image analysis bias ( Figure 3).

In conclusion, we have screened TDP-43 commercial antibodies by Western Blot, immunoprecipitation and immunofluorescence and identified several high-performing antibodies under our standardized experimental conditions.

## Methods

### Antibodies

All TDP-43 antibodies are listed in Table 2, together with their corresponding Research Resource Identifiers (RRID), to ensure the antibodies are cited properly. ^10^ Peroxidase-conjugated goat anti-mouse and anti-rabbit antibodies are from Thermo Fisher Scientific (cat. number 62-6520 and 65-6120). Alexa-555-conjugated goat anti-mouse and anti-rabbit secondary antibodies are from Thermo Fisher Scientific (cat. number A21424 and A21429).

**Table 2.** Summary of the TDP-43 antibodies tested.

Company	Catalog number	Lot number	RRID (Antibody Registry)	Clonality	Clone ID	Host	Concentration (μg/μl)	Vendors recommended applications
Proteintech	10782-2-AP	97534	AB_615042	polyclonal	-	rabbit	0.60	Wb, IF
Proteintech	12892-1-AP	94163	AB_2200505	polyclonal	-	rabbit	0.65	Wb, IP, IF
Proteintech	80001-1-RR **	23000002	AB_2882933	recombinant-mono	11N20	rabbit	0.25	Wb
Proteintech	80002-1-RR **	23000003	AB_2882934	recombinant-mono	16A22	rabbit	0.25	IF
Bio-Techne	MAB7778 *	CHGW0121061	AB_2920573 ^1^	monoclonal	671834	mouse	0.50	Wb
Bio-Techne	NBP1-92695 *	122117	AB_11005586	monoclonal	3H8	mouse	1.00	Wb, IF
Thermo Fisher Scientific	711051 **	2352341	AB_2633110	recombinant-poly	1HCLC	rabbit	0.50	Wb, IP, IF
Thermo Fisher Scientific	MA5-27828 *	XB3501489	AB_2735390	monoclonal	GT733	mouse	1.00	Wb, IF
Thermo Fisher Scientific	MA5-32627 **	XB3501035	AB_2809904	recombinant-mono	JM51-10	rabbit	1.00	Wb, IP, IF
ABclonal	A19123 **	4000000492	AB_2862616	recombinant-mono	ARC0492	rabbit	0.93	Wb, IF
Cell Signaling Technology	89789 **	1	AB_2800143	recombinant-mono	D9R3L	rabbit	0.003	Wb, IF
Cell Signaling Technology	89718 **	1	AB_2920572 ^1^	recombinant-mono	E2G6G	rabbit	0.014	Wb
GeneTex	GTX630196 *	41505	AB_2888198	monoclonal	GT225	mouse	1.00	Wb, IF
GeneTex	GTX630197 *	41505	AB_2888199	monoclonal	GT733	mouse	1.00	Wb, IF
Abcam	ab109535 **	GR3324454-4	AB_10859634	recombinant-mono	EPR5810	rabbit	0.03	Wb, IF-Methanol
Abcam	ab133547 **	GR3345629-4	AB_2920621 ^1^	recombinant-mono	EPR5811	rabbit	0.67	Wb, IF
Abcam	ab190963 **	GR233962-6	AB_2920622 ^1^	recombinant-mono	EPR18554	rabbit	0.79	Wb, IP, IF
Abcam	ab254166 **	GR3316998-3	AB_2920620 ^1^	recombinant-mono	DB9	mouse	0.47	Wb

Wb=Western blot; IF=immunofluorescence; IP=immunoprecipitation.

* Monoclonal antibody.

** Recombinant antibody.

^1^ Refer to RRID recently added to the Antibody Registry (on January 2023), they will be available on the Registry website in coming weeks.

### Cell culture

Both HAP1 WT and *TARDBP* KO cell lines used are listed in Table 1, together with their corresponding RRID, to ensure the cell lines are cited properly. ^11^ Cells were cultured in DMEM high glucose (GE Healthcare cat. number SH30081.01) containing 10% fetal bovine serum (Wisent, cat. number 080450), 2 mM L-glutamate (Wisent, cat. number 609065), 100 IU penicillin and 100 μg/ml streptomycin (Wisent cat. number 450201).

### Antibody screening by Western blot

Western blots were performed as described in our standard operating procedure. ^12^ HAP1 WT and *TARDBP* KO were collected in RIPA buffer (25mM Tris-HCl pH 7.6, 150mM NaCl, 1% NP-40, 1% sodium deoxycholate, 0.1% SDS) from Thermo Fisher Scientific (cat. number 0089901), supplemented with protease inhibitor from MilliporeSigma (cat. number P8340). Lysates were sonicated briefly and incubated for 30 min on ice. Lysates were spun at ~110,000 x g for 15 min at 4°C and equal protein aliquots of the supernatants were analyzed by SDS-PAGE and Western blot. BLUelf prestained protein ladder from GeneDireX (cat. number PM008-0500) was used.

Western blots were performed with precast midi 4-20% Tris-Glycine polyacrylamide gels from Thermo Fisher Scientific (cat. number WXP42012BOX) and transferred on nitrocellulose membranes. Proteins on the blots were visualized with Ponceau staining which is scanned to show together with individual Western blots. Blots were blocked with 5% milk for 1 hr, and antibodies were incubated overnight at 4°C with 5% bovine serum albumin (BSA) (Wisent, cat number 800-095) in TBS with 0,1% Tween 20 (TBST) (Cell Signaling Technology, cat. number 9997). Following three washes with TBST, the peroxidase conjugated secondary antibody was incubated at a dilution of ~0.2 μg/ml in TBST with 5% milk for 1 hr at room temperature followed by three washes with TBST. Membranes were incubated with ECL (Thermo Fisher Scientific, cat. number 32106) prior to detection with the iBright™ CL1500 Imaging System (Thermo Fisher Scientific, cat. number A44240).

### Antibody screening by immunoprecipitation

Immunoprecipitation was performed as described in our standard operating procedure. ^13^ Antibody-bead conjugates were prepared by adding 2 μg to 500 μl of Pierce IP Lysis Buffer from Thermo Fisher Scientific (cat. number 87788) in a 1.5 ml microcentrifuge tube, together with 30 μl of Dynabeads protein A- (for rabbit antibodies) or protein G- (for mouse antibodies) from Thermo Fisher Scientific (cat. number 10002D and 10004D, respectively). Tubes were rocked for ~2 hrs at 4°C followed by two washes to remove unbound antibodies.

HAP1 WT were collected in Pierce IP buffer (25 mM Tris-HCl pH 7.4, 150 mM NaCl, 1 mM EDTA, 1% NP-40 and 5% glycerol) (Thermo Fisher Scientific, cat. number 87788), supplemented with protease inhibitor (Millipore Sigma, cat. number P8340). Lysates were rocked for 30 min at 4°C and spun at 110,000× g for 15 min at 4°C. 0.5 ml aliquots at 2.0 mg/ml of lysate were incubated with an antibody-bead conjugate for ~2 hrs at 4°C. The unbound fractions were collected, and beads were subsequently washed three times with 1.0 ml of IP lysis buffer and processed for SDS-PAGE and Western blot on precast midi 4-20% Tris-Glycine polyacrylamide gels. Prot-A: HRP (MilliporeSigma, cat. number P8651) was used as a secondary detection system at a dilution of 0.4 μg/ml for an experiment where a rabbit antibody was used for both immunoprecipitation and its corresponding Western blot.

### Antibody screening by immunofluorescence

Immunofluorescence was performed as described in our standard operating procedure. ^9^ HAP1 WT and *TARDBP* KO were labelled with CellTracker ^TM^ green (Thermo Fisher Scientific, cat. number C2925) or CellTracker ^TM^ deep red (Thermo Fisher Scientific, cat. number C34565) fluorescence dye, respectively. The nuclei were labelled with DAPI (Thermo Fisher Scientific, cat. number D3571) fluorescent stain. WT and KO cells were plated on glass coverslips as a mosaic and incubated for 24 hrs in a cell culture incubator at 37°C, 5% CO _2_. Cells were fixed in 4% paraformaldehyde (PFA) (Beantown chemical, cat. number 140770-10 ml) in phosphate buffered saline (PBS) (Wisent, cat. number 311-010-CL) for 15 min at room temperature and then washed 3 times with PBS. Cells were permeabilized in PBS with 0,1% Triton X-100 (Thermo Fisher Scientific, cat. number BP151-500) for 10 min at room temperature and blocked with PBS with 5% BSA, 5% goat serum (Gibco, cat. no 16210-064) and 0.01% Triton X-100 for 30 min at room temperature. Cells were incubated with IF buffer (PBS, 5% BSA, 0,01% Triton X-100) containing the primary TDP-43 antibodies overnight at 4°C. Cells were then washed 3 × 10 min with IF buffer and incubated with corresponding Alexa Fluor 555-conjugated secondary antibodies in IF buffer at a dilution of 1.0 μg/ml for 1 hr at room temperature with DAPI. Cells were washed 3 × 10 min with IF buffer and once with PBS.

Images were acquired on an ImageXpress micro widefield high-content microscopy system (Molecular Devices), using a 20×/0.45 NA air objective lens and scientific CMOS camera (16-bit, 1.97 mm field of view), equipped with 395, 475, 555 and 635 nm solid state LED lights (Lumencor Aura III light engine) and bandpass emission filters (432/36 nm, 520/35 nm, 600/37 nm and 692/40 nm) to excite and capture fluorescence emission for DAPI, CellTracker ^TM^ Green, Alexa fluor 555 and CellTracker ^TM^ Red, respectively. Images had pixel sizes of 0.68 × 0.68 microns. Exposure time was set with maximal (relevant) pixel intensity ~80% of dynamic range and verified on multiple wells before acquisition. Since the IF staining varied depending on the primary antibody used, the exposure time was set using the most intensely stained well as reference. Frequently, the focal plane varied slightly within a single field of view. To remedy this issue, a stack of three images per channel was acquired at a z-interval of 4 microns per field and best focus projections were generated during the acquisition (MetaExpress v6.7.1, Molecular Devices). Segmentation was carried out on the projections of CellTracker ^TM^ channels using CellPose v1.0 on green (WT) and far-red (KO) channels, using as parameters the ‘cyto’ model to detect whole cells, and using an estimated diameter tested for each cell type, between 15 and 20 microns. ^14^ Masks were used to generate cell outlines for intensity quantification. Figures were assembled with Adobe Illustrator.

## Data Availability

Zenodo: Antibody Characterization Report for TDP-43, https://doi.org/10.5281/zenodo.7249802. ^15^ Zenodo: Dataset for the TDP-43 antibody screening study, https://doi.org/10.5281/zenodo.7665963. ^16^ Data are available under the terms of the Creative Commons Attribution 4.0 International license (CC-BY 4.0).
