# Supplementary material for: The identification of high-performing antibodies for TDP-43 for use in Western Blot, immunoprecipitation and immunofluorescence
Source: F1000Res. 2023 Jun 20;12:277. Originally published 2023 Mar 14. [Version 2] doi: 10.12688/f1000research.131852.2 (PMC10285334; doi:10.12688/f1000research.131852.2)
Supplement: Peer review history

# Peer review: Reviewer response for version 1

**DOI:** 10.5256/f1000research.144735.r178389

**Published:** 2023-06-14

**Authors:** Seiji Watanabe

**Affiliations:** 1 Department of Neuroscience and Pathobiology, Research Institute of Environmental Medicine, Nagoya University, Nagoya, Aichi Prefecture, Japan

Various antibodies against TDP-43 are now commercially available from several companies, but their validation is still insufficient. This study comprehensively validated the performance of various antibodies, which will provide important information for researchers when they select TDP-43 antibodies for their studies. The procedures are detailed and carefully described, and together with the references, are well reproducible.

 Pathological TDP-43 is generally aggregated, fragmented, and hyperphosphorylated, which may affect antibody recognition. Thus, although it would be beyond the scope of this study, future validation using human patients’ samples, as recommended by the reviewer #1, or of the recognition sites of each antibody would make this data even more valuable.

 The references 9, 12, and 13 were found to be available on Zendo, but it is difficult to reach them because of a lack of direct links. It is recommended that the DOI be appended.

Are sufficient details of methods and materials provided to allow replication by others?

Yes

Is the rationale for creating the dataset(s) clearly described?

Yes

Are the datasets clearly presented in a useable and accessible format?

Yes

Are the protocols appropriate and is the work technically sound?

Yes

Reviewer Expertise:

Amyotrophic Lateral Sclerosis, Protein Aggregation, Organelle Contact

I confirm that I have read this submission and believe that I have an appropriate level of expertise to confirm that it is of an acceptable scientific standard.

# Peer review: Reviewer response for version 1

**DOI:** 10.5256/f1000research.144735.r166439

**Published:** 2023-03-20

**Authors:** Makoto Urushitani

**Affiliations:** 1 Department of Neurology, Shiga University of Medical Science, Otsu, Japan

Due to the continuing significance of morphological assay to clarify TDP-43 proteinopathy, the characteristics of the antibody are crucial to drawing convincing and replicable results. Numerous antibodies against TDP-43 are commercially available to meet the needs. As is often the case, however, the results could be misleading unless the antibodies are used with a sufficient understanding of their properties. However, such information is hardly accessible until our use.

 In this work, the authors comprehensively investigated the specificity and affinity of commercially available antibodies against TDP-43 by analyzing Western blotting, immunoprecipitation, and immunofluorescence. Another advantage of their work is adopting HAP1 cells through careful screening from transcription levels and TARDBP-KO cells. They successfully suggested several antibodies, which are conformation- and sequence-specific with high reactivity. Experimental protocols are clearly written, and the data presentation is compelling. This work deserves considerable attention because such information may help researchers minimize the time for optimization and acquire solid and replicable results. The weak points of their work is a lack of experiments using ALS-linked TDP-43 mutations or immunohistochemistry of ALS patients. However, those are beyond their scope, which awaits future validation.

Are sufficient details of methods and materials provided to allow replication by others?

Yes

Is the rationale for creating the dataset(s) clearly described?

Yes

Are the datasets clearly presented in a useable and accessible format?

Yes

Are the protocols appropriate and is the work technically sound?

Yes

Reviewer Expertise:

Motor neuron disease, protein misfolding, antibody generation

I confirm that I have read this submission and believe that I have an appropriate level of expertise to confirm that it is of an acceptable scientific standard.

# Response

**Published:** 2023-03-21

**Authors:** Kathleen Southern

**Affiliations:** McGill University, Canada

Thank you to Makoto Urushitani for your review of our publication. Although there is an abundance of commercial antibodies available for TDP-43, there lacks guidance to help researchers find the appropriate antibody for their experimental need.  Accordingly, we too agree that the characterization of antibodies is a determinant factor when drawing convincing and replicable results. As such, the YCharOS initiative has set out to solve the antibody reproducibility crisis by characterizing commercially available antibodies for human proteins.

 In terms of scoring the antibodies based on performance, we have found that for the most part, scientists interested in our reports have the expertise to interpret the antibody characterization data. Moreover, because we tested the antibodies under one set of conditions, and the scoring/recommendation would be valid only under this precise experimental setup and in the cell line used. That said, YCharOS reports serve as an invaluable guide pointing scientists to appropriate antibodies for their experimental needs.

 In the future, we hope to study the ALS protein-network in ALS patients, using the YCharOS reports as a guide to select the appropriate antibodies. As for now, our aim for this publication is to assist researchers in selecting high-quality TDP-43 antibodies for studying TDP-43 proteinopathies.
